# Prediction of G Protein-Coupled Receptors With CTDC Extraction and MRMD2.0 Dimension-Reduction Methods

**DOI:** 10.3389/fbioe.2020.00635

**Published:** 2020-06-25

**Authors:** Xingyue Gu, Zhihua Chen, Donghua Wang

**Affiliations:** ^1^Institute of Computing Science and Technology, Guangzhou University, Guangzhou, China; ^2^Department of General Surgery, Heilongjiang Province Land Reclamation Headquarters General Hospital, Harbin, China

**Keywords:** feature extraction, CTD, MRMD2.0, Matplotlib, predict GPCRs

## Abstract

The G Protein-Coupled Receptor (GPCR) family consists of more than 800 different members. In this article, we attempt to use the physicochemical properties of Composition, Transition, Distribution (CTD) to represent GPCRs. The dimensionality reduction method of MRMD2.0 filters the physicochemical properties of GPCR redundancy. Matplotlib plots the coordinates to distinguish GPCRs from other protein sequences. The chart data show a clear distinction effect, and there is a well-defined boundary between the two. The experimental results show that our method can predict GPCRs.

## Introduction

G protein-coupled receptors (GPCRs) are the largest receptor superfamily. According to their sequence similarity, they are divided into 6 subfamilies (AF), of which the Rhodopsin or rhodopsin-like family is the largest and most widely studied family (Fredriksson et al., 2003; Liu and Zhu, 2019; Ru et al., 2020). Class A has approximately 284 members in humans, and Class B subfamilies can be further divided into two unused families: Class B1, named secretin, secrete protein-like receptors, and Class B2 (adhesion) adhere to GPCRs. Class B1 and Class B2 contain 15 members and 33 members in humans, respectively. The adhesive G protein-coupled receptor (ADGR) family is one of the oldest GPCR families. It exists in primitive animals, and even in several basic fungi, and is the ancestor of the B1 subfamily of GPCRs (Nordstrm et al., 2009; Krishnan et al., 2012). Finally, the class C glutamate family is composed of peptide receptors. The class F frizzled protein family has appsroximately 11 members in humans.

Protein classification is one of the key issues in bioinformatics and plays an important role in the identification and study of gene markers (Tibshirani, 1996; Cheng and Hu, 2018; Feng, 2019; Guo et al., 2019). With the development of machine learning, protein classification and prediction have entered a new era. Machine learning can use previous experience and data to automatically improve the performance of algorithms, build appropriate models, and discriminate new protein sequences. Islam et al. (2017) applied a natural language processing N-Gram model to classify proteins. The above machine learning methods have achieved certain effects in protein classification. This article uses feature extraction and dimension reduction of GPCR proteins to distinguish between the properties of the extracted proteins. Finally, Matplotlab is used to distinguish GPCRs from non-GPCRs. In the article Prediction of G Protein-Coupled Receptors (Liao et al., 2016), the 188D method is used to extract the protein features, and then cross validation and random forest are used to accurately divide the GPCR and non-gpcr protein sequences. In this paper, the CTD mode (Zou et al., 2013) is used, where C represents the content of each hydrophobic amino acid, T represents the frequency of the divalent peptide, and D represents the amino acid distribution at the five positions of the sequence. After using CTDC feature extraction method, the innovative feature of this experiment is that the redundant features are well-extracted using dimensionality reduction. Finally, the machine learning method and Matplotlib are used to draw a graph that distinguishes GPCRs from non-GPCRs.

## Materials and Methods

### Datasets

1. The original 5027 G protein-coupled receptors (GPCRs) were obtained in fasta format from the database (http://www.UniProt.org/); 2. The initial sequence was pre-processed using the protein clustering programme CDHIT (http://cd-hit.org/) to improve the analysis performance and reduce the homology of the predicted sequence (Zou et al., 2020). The critical value of sequence identity was located at 0.8. Finally, 2,495 GPCR sequences were obtained from the positive data set. 3. The positive sequences of all the protein sequences were removed, and 10,386 non-GPCR protein sequences were produced as the positive dataset (Liao et al., 2016).

### Feature Extraction Methods

#### Principle

CTD represents the composition, transition, and distribution, respectively. Its principle is to replace the amino acid sequence with mathematical symbols representing physical and chemical properties (Cheng et al., 2018a). Because the protein sequence information is of different lengths, CTD is used to obtain fixed-length information from proteins as input to machine learning. In protein or peptide sequences, CTD represents physicochemical properties or amino acid distribution patterns of specific structures (Dubchak et al., 1995, 1999; Cai et al., 2003; Zhang et al., 2011; Ding et al., 2017). These features are very important for protein sequence analysis (Wei et al., 2018; Liu et al., 2019; Liu et al., 2019a; Yan et al., 2019; Chen et al., 2020). According to the main amino acid indicators of Tomii and Kanehisa (Kentaro and Minoru, 1996), amino acids are divided into three groups according to seven physical and chemical properties, as shown in Table 1.

**Table 1:** Seven types of physicochemical properties and the division of amino acids.

**Seven types of physicochemical properties**	**Division: 1**	**Division: 2**	**Division: 3**
Secondary structure; Amino acids	Helix; M, E, A, K, R, H, L, Q	Strand; W, F, T, V, I, Y, C	Coil; S, D, G, P, N
Hydrophobicity; Amino acids	Polar; N, Q, D, E, K, R	Neutral; Y, P, H, S, T, A, G	Hydrophobicity; M, F, I, L, C, W, V
Normalized van der Waals volume; Amino acids	0–2.78; T, S, P, A, G, D	2.95–94.0; Q, L, V, N, E, I	4.03–8.08; M, H, K, F, R, Y, W
Solvent accessibility; Amino acids	Buried; W, V, I, C, G, F, A, L	Exposed; Q, E, D, N, K, P	Intermediate; H, Y, M, S, P, T
Polarizability; Amino acids	0–1.08; G, A, S, D, T	0.128–120.186; G, P, N, V, E, Q, I, L	0.219–0.409; K, M, H, F, R, Y, W
Charge; Amino acids	Positive; K, R	Neutral; Q, G, H, I, A, N, C, L, M, FP, S, T, W, Y, V	Negative; E, D
Polarity; Amino acids	4.9–6.2; L, I, F, W, C, M, V, Y	8.0–9.2; P, A, T, G, S	10.4–13.0; H, Q, R, K, N, E, D

CTD (Dubchak et al., 1999) is very helpful for enzyme prediction. Composition (Cai et al., 2003; Han et al., 2004; Chen W. et al., 2019; Liu, 2019) refers to the number of specific amino acids in a protein sequence divided by the total length N of the amino acid in the protein sequence:

(i) $$\begin{array}{l} \text{Composition}\left(\text{e}\right)=\frac{n_{e}}{N} \end{array}$$

where *n*_*e*_ represents the sum of the number of e, a particular amino acid, in the sequence. e could be 1, 2, or 3, which represents the type of amino acid.

Assuming two specific amino acids are a and b, transition (T) means the number of ab and ba divided by the length of the protein sequence N-1:

(ii) $$\begin{array}{l} \text{Transition}\left(\text{ab}+\text{ba}\right)=\frac{n_{ab}+n_{ba}}{N-1} \end{array}$$

The distribution is the position of a specific amino acid in the protein/the total length of the protein sequence, which represents the chain length at which the first, 25, 50, 100% amino acids of this particular amino acid are located.

**Figure 1:**
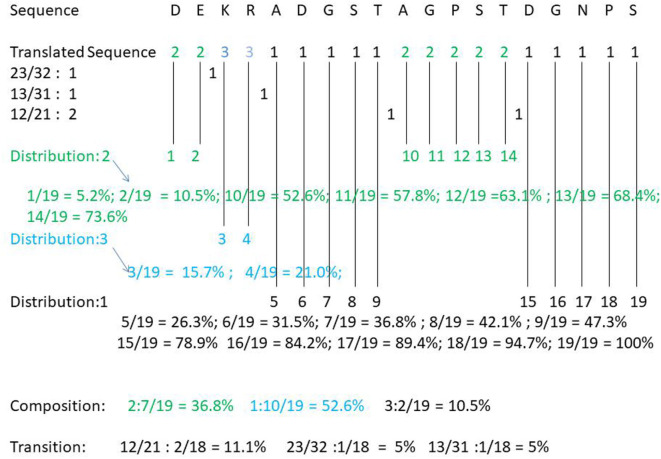
Computational flow of CTD eigenvectors in protein sequences.

For example, take the following protein sequence: DEKRADGSTAGPSTDGNPS. According to Table 1, DE is the amino acid sequence of classification 2 under Charge, KR is the amino acid sequence of category 3 under Charge, and ADGST is the amino acid sequence of classification 1 under Polarizability. AGPST is an amino acid sequence of Polarity 2, and DGNPS is the amino acid sequence of classification 1 under the Secondary Structure. Thus, our protein sequence is converted by CTD to 2233111112222211111. The following shows how the protein sequence Composition, Transition, Distribution is calculated (see Figure 1).

Composition of category 2: 7/(7 + 2 + 10 = 19)= 36.8%; Composition of category 3: 2/19 = 10.5%; Composition of category 1: 10/19 = 52.6%. Transition (23, 32) = 1/18 = 5.5%; Transition (12, 21) = 2/18 = 11.1%; Transition (13, 31) = 1/18 = 5.5%. Distribution (1) = 5/19, 6/19, 7/19, 8/19, 15/19, 16/19, 17/19, 18/19, 19/19; Distribution (2) =1/19, 2/19, 10/19, 11/19, 12/19, 13/19, 14/19; Distribution 3 is equal to 3/19, 4/19. The final CTD results of DEKRADGSTAGPSTDGNPS are as follows: Composition (2): 36.8%, Composition (3): 10.5%, Composition (1): 52.6%. T (23, 32): 5.5%, T (12, 21): 11.1%, T (13, 31): 5.5%; D (1): 26.3, 31.5, 36.8, 42.1, 78.9, 84.2, 89.4, 94.7, 100%; D (2): 5.2, 10.5, 52.6, 57.8, 63.1, 68.4, 73.6%; D (3): 15.7, 21.0%.

### Dimensionality Reduction

**Figure 2:**
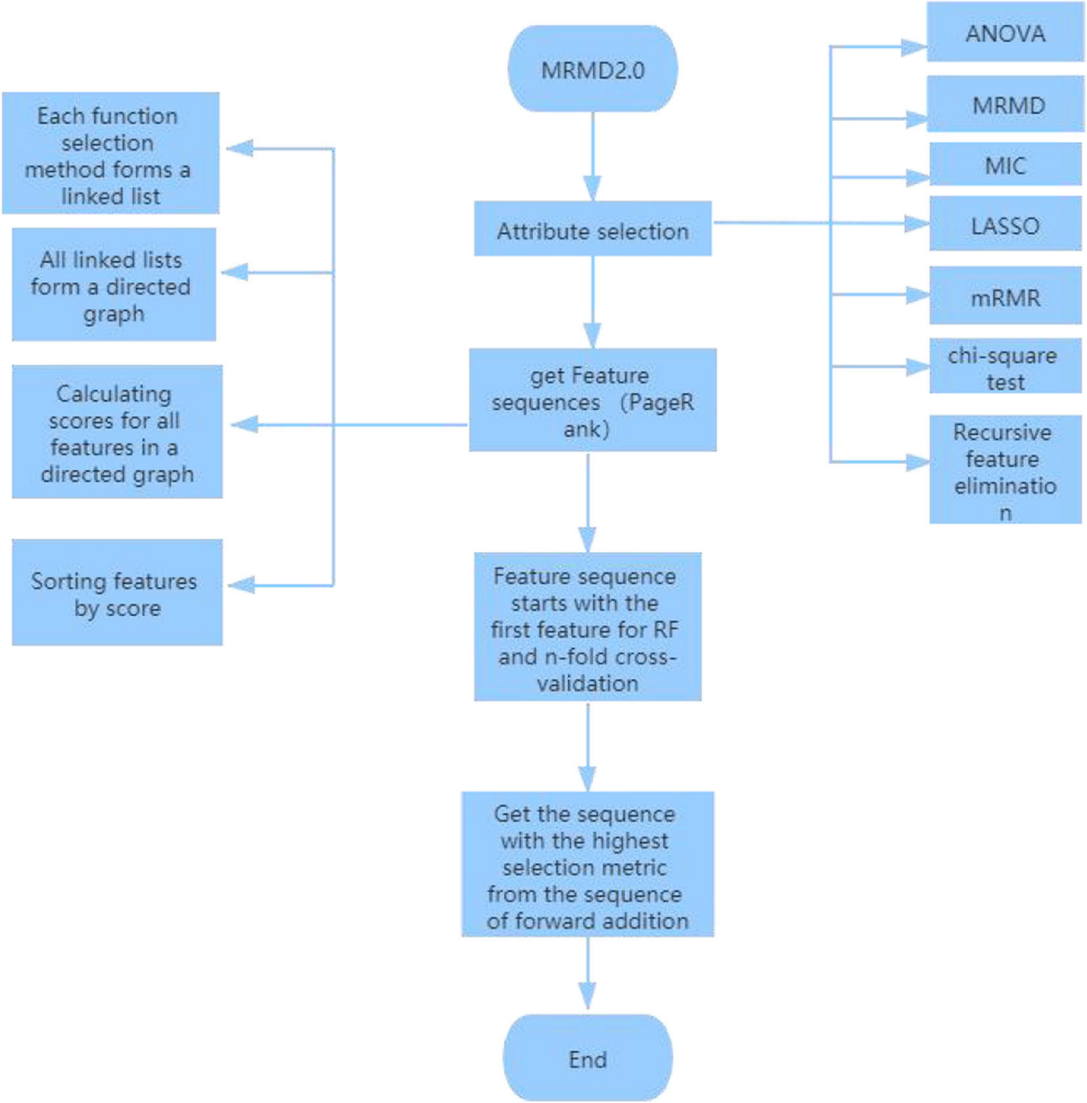
The specific dimension reduction process.

The MRMD2.0 (Wei et al., 2015; Zou et al., 2016a,b) algorithm is used to reduce the dimensions of the files after using CTDC to extract features. The specific process of dimensionality reduction is:

1. Attribute selection: Using analysis of variance to test the significance of the difference between the mean values of two or more samples; maximum correlation and maximum distance MRMD feature classification and accuracy and stability of prediction tasks; MIC is based on a non-parametric information-based maximum parameter exploration for measuring the linear or non-linear strength of two variables X and Y; the minimum absolute contraction and selection operator (LASSO) (Tibshirani, 1996; Guo et al., 2019) uses an L1 regularized linear regression method; Minimal Redundancy-Maximum Correlation (mRMR) method expands the representativeness of a feature set by requiring features to be maximally different from each other; chi-square test is a widely used hypothesis test based on the chi-square distribution for common hypothesis testing; Recursive Feature Elimination (RFE) classifies data according to the size of the correlation coefficients or importance of feature attributes. Through recursive elimination of functions in each cycle, RFE attempts to eliminate possible dependencies and collinearity in the model.
2. Function ranking PageRank algorithm: In the attribute selection method used above, point a to b because feature b is more important than feature a. Finally, the result of each function selection method forms a link list. Using the PageRank algorithm to rank these links, a directed graph is formed, and each feature receives a score. A ranking is then obtained according to the level of the feature, a, b, c, d, e .
3. Finally, choose the best outcome of the sequence. Since the first feature “a” in the new sequence has the highest score, random forest (Pang et al., 2006; Ding et al., 2016; Cheng et al., 2018b; Liu et al., 2019b; Su et al., 2019; Wei et al., 2019; Xu et al., 2019c; Lv et al., 2020) is used for 5-fold cross-validation starting from the first feature. The highest standard score is made by comparing the three sequences: “a,” “a,b;” “a,b,c,d,e.” Finally, five data indicators were used: f-score, precision, recall, MCC and AUC (Xu et al., 2018a; Cheng, 2019; Cheng L. et al., 2019; Ding et al., 2019; Zeng et al., 2019a, 2020; Zhang et al., 2019; Liu and Chen, 2020; Wang et al., 2020), and the sequence with the highest index and the highest score for dimension reduction was found. The specific dimension reduction process is shown in Figure 2.

### Algorithm Steps

**Figure 3:**
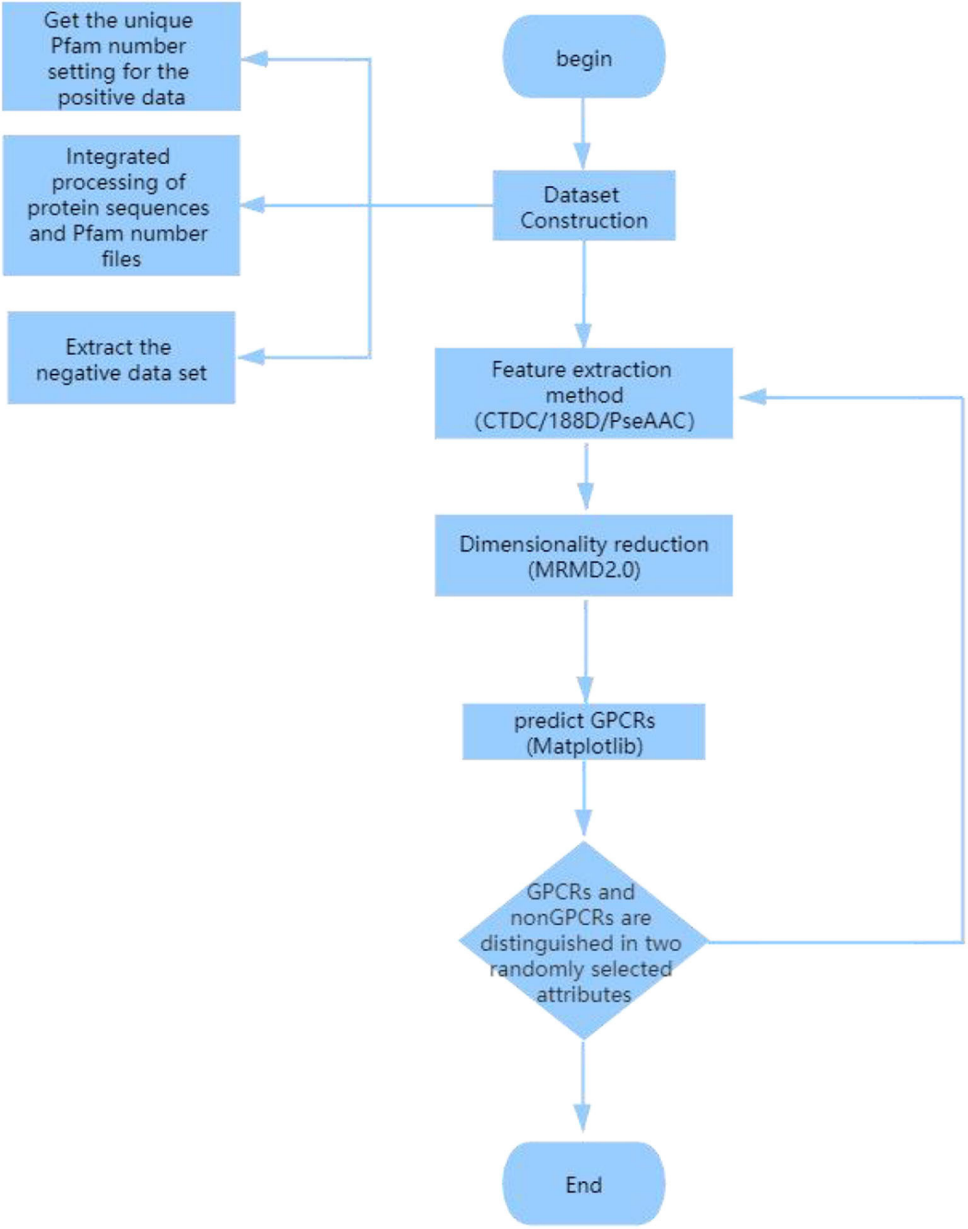
Experimental flow chart for prediction of G protein-coupled receptors.

GPCR sequence protein features are extracted using specific protein extraction methods. Any two attributes in the extracted features are divided into GPCRs and non-GPCRs. Finally, Matplotlib is used to divide any two attributes in the extracted features into GPCRs and non-GPCRs (the experimental flow chart is shown in Figure 3):

1. Using all the different positive protein samples, extract the corresponding Pfam protein sequence from the “family and domain” of the UniProt website and delete the redundant and identical Pfam number. Then, the unique Pfam number obtained for the positive data set (Liao et al., 2016).
2. All the protein sequences are integrated into the Pfam number file, and the protein sequences with the same Pfam sequence are then merged into the same file named after the Pfam number.
3. Delete the files with a positive Pfam number. In the remaining Pfam number files, the negative data set (Liao et al., 2016) is extracted from the longest sequence of each Pfam.
4. Use the CTDC method command to extract specific features in fasta files to generate GPCRs and non-GPCRs .csv files; positive GPCRs sample are marked as 0, negative sample are marked as −1, and the GPCRs and non-GPCRs .csv files are combined into one file.
5. The combined .csv file was reduced by MRMD2.0, and the reduced CTDC-mRMD2.0.csv file was obtained.
6. Select any two attributes of the 39 attributes in the CTDC sequence. GPCRs are purple and marked 0, and non-GPCRs are green and marked 1; Using Matplotlib, plot the picture of GPCRs and non-GPCRs.

## Results

### Comparison of Effects of Different Features

**Figure 4:**
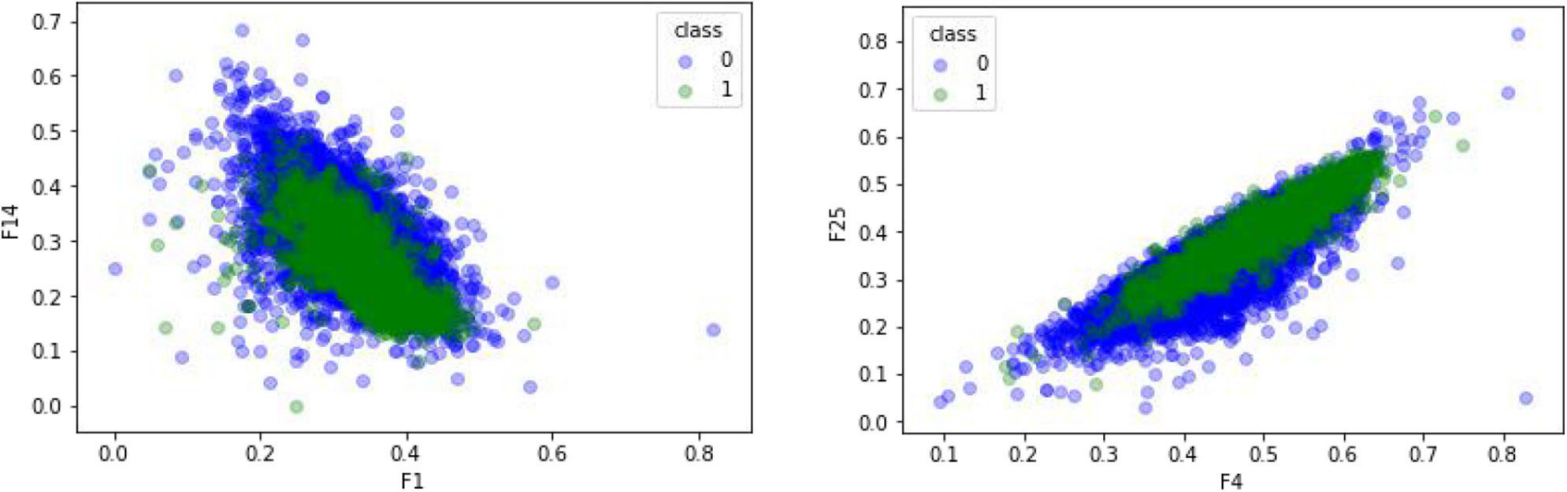
Comparison of effects of different features.

CTDC was used to extract the characteristics of the GPCR protein feature sequences sample, including 39 properties. Previous studies showed that feature extraction is very important for constructing the computational predictors (Wei et al., 2017a,b; Xu et al., 2018b; Liang et al., 2019; Liu and Li, 2019; Patil and Chouhan, 2019; Shen et al., 2019; Zhang and Liu, 2019; Junwei et al., 2020; Liu et al., 2020; Wen et al., 2020). Any two of the 39 attributes were selected and plotted using Matplotlab to obtain the sample differentiation graph of GPCRs and non-GPCRs, as shown in Figure 4. Among them, the abscissa and the ordinate in the chart represent two of the 39 attributes. The x-coordinate of Figure 4 on the left is the first of the 39 properties, “hydrophobicity_PRAM900101,” named “RKEDQN,” which is hydrophilic. The y-coordinate is the 14th property, “hydrophobicity_PRAM900101,” named “GASTPHY,” which is neutral. In the right diagram of Figure 4, the X coordinate is the fourth attribute in the CTDC feature extraction method, normwaalsvolume: NVEQIL. The Y coordinate is the 25th attribute in CTDC, hydrophobicity_ENGD860101: CVLIMF. As seen from the chart, GPCRs and non-GRCRs are represented by blue and green, respectively, in which GPCRs and non-GPCRs can be clearly distinguished.

### Comparison of Different Feature Extraction Methods

**Figure 5:**
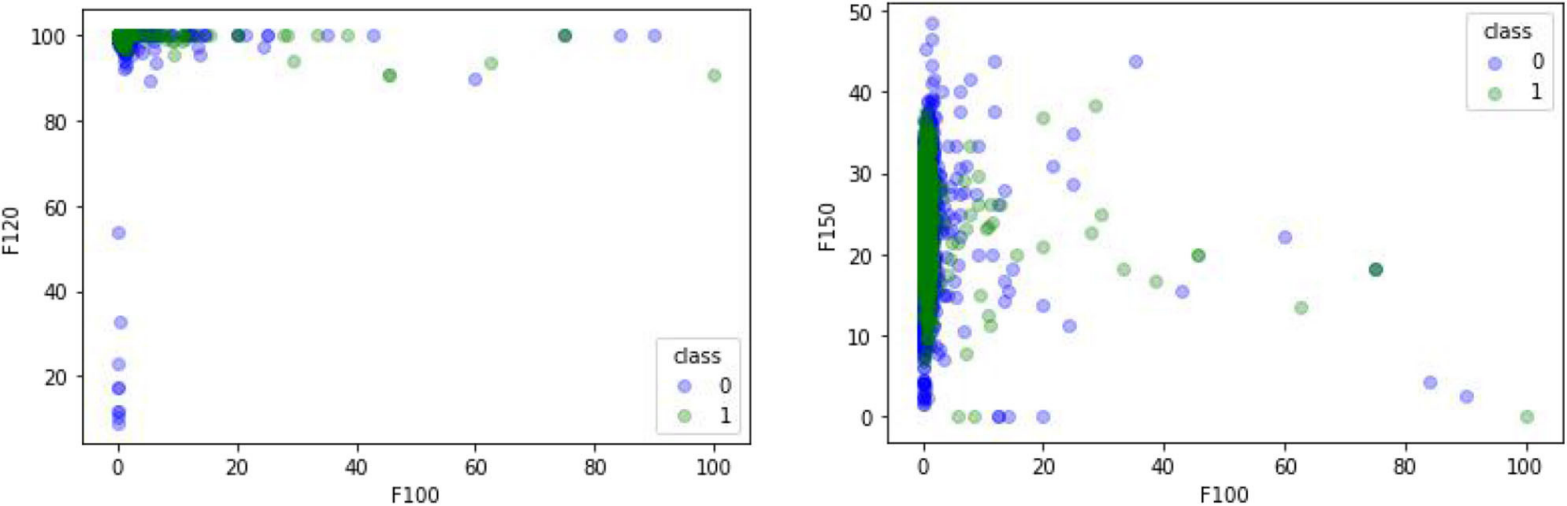
Comparison of different feature extraction methods.

A comparative experiment was conducted, and the GPCR protein feature sequences are extracted by the 188D feature extraction method. The experimental effect is shown in Figure 5. In Figure 5, 120 and 100 dimensions of 188D are used. Non-GPCRs and GPCRs are marked as −1 and 1, respectively. It can be seen from the chart that the differentiation effect of GPCRs and non-GPCRs is very poor, but the differentiation effect of Figure 4 is very good. Thus, whether GPCRs and non-GPCRs can be distinguished well is related to the selected feature extraction method.

### Comparison of Results of Different Dimensionality Reduction Methods

**Figure 6:**
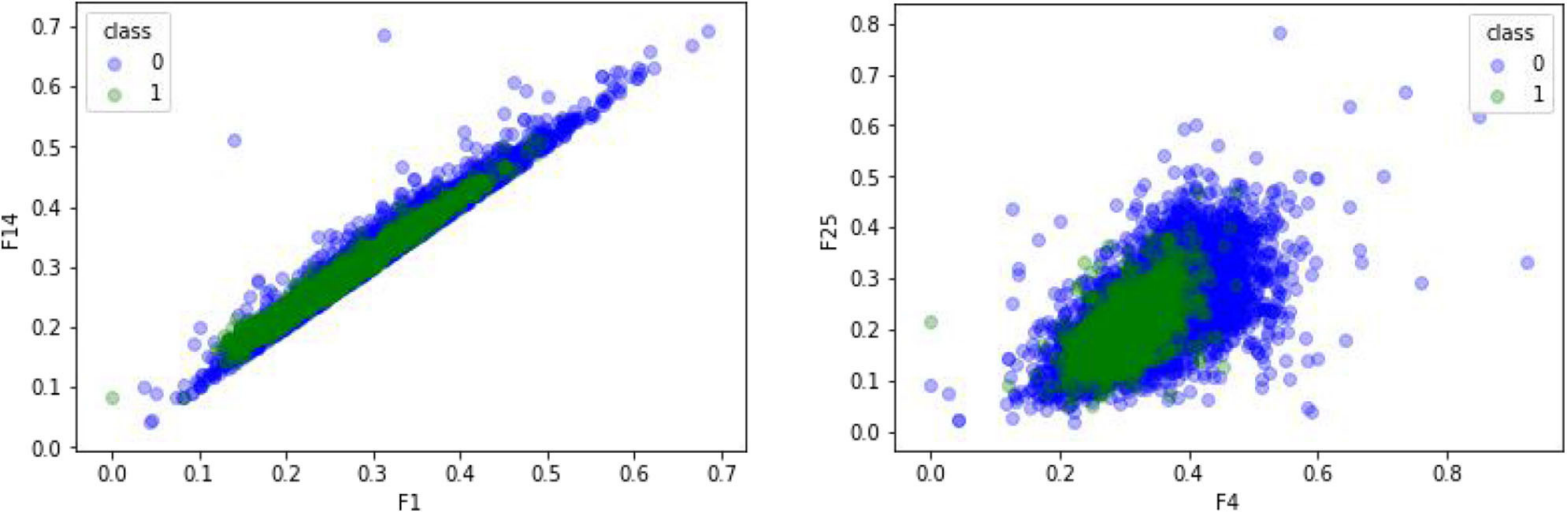
Comparison of results of different dimensionality reduction methods.

The feature sequences of GPCR protein are extracted by the mRMR (Ding and Peng, 2005; Peng et al., 2005; Wang et al., 2018) dimensionality reduction method. 0 represents negative sample non-GPCRs, and 1 represents positive sample GPCRs. The experimental results are shown in Figure 6. In comparison with Figure 4, the two figures adopt the same feature extraction method of CTDC, the same attribute features and different dimension reduction methods. As seen from the figure, the difference between GPCRs and non-GPCRs was also very high after the dimension reduction method was used, and positive and negative samples are clearly distinguished.

### Comparison With Others

In the study of Prediction of G Protein-Coupled Receptors with SVM-Prot Features and Random Forest (Liao et al., 2016), the researchers adopted a method different from the method in this paper to predict GPCRs and non-GPCRs. The experimental steps they adopted were as follows: 1. Extract GPCR and non-GPCR sample characteristics with 188D (Balfanz et al., 2013) 2. The sample sequences were divided into five parts, four of which were for the training set and the remaining one for the test set. In these four parts, positive and negative samples were treated with a strike balance 3. Random Forest was applied to the training samples, and the accuracy of the test samples was measured 4. Finally, Sn, Sp, Acc, MCC, and AUC standards were adopted to measure the accuracy. The correct classification rate of the five independent test sets was 90.64, 90.37, 88.04, 93.28, and 95.73, with an average rate of 91.61 ± 2.96%.

## Conclusion

With the feature extraction method of CTDC, GPCRs and non-GPCRs can be well-distinguished from the two randomly selected dimensions. The same CTDC feature extraction method was used, but another dimension reduction method, mRMR, was selected. Compared with mRMD2.0, the differentiation effect was similar, and GPCRs and non-GPCRs could be significantly predicted. Using different feature extraction methods (188D) and the same dimensionality reduction method (mRMD2.0), GPCRs and non-GPCRs had no clear dividing line. In conclusion, different methods of feature extraction and the same method of dimensionality reduction have different effects on GPCRs and non-GPCRs. Therefore, the feature extraction method is the direct factor for distinguishing GPCRs from non-GPCRs.

However, a similar work was done in the Prediction of G protein-coupled sensor (Nordstrm et al., 2009) study. Compared with our study, the defects were as follows: 1. The 188D feature extraction method with more dimensions was adopted, the 188D feature extraction method had more feature dimensions, and the feature information of proteins was more complete and more comprehensive. The dimension information extracted by the CTDC method in this experiment has only 39 attribute characteristics, and there are less data. In addition, there is less redundant information after dimension reduction. 2. Five independent test sets and training sets were divided in the Prediction of G protein-coupled sensor study, and the positive and negative samples in the training set tended to be balanced by the use of strike. However, defects in the strike method lead to inaccuracy of the data. In this paper, on the basis of original data collection, feature extraction and dimensionality reduction were directly carried out to distinguish GPCRs sample from non-GPCRs sample to obtain more accurate prediction results. Compared with this paper, the advantages are as follows: 1. The accuracy of the Prediction of G Protein by Coupled sensor study is approximately 90%; while the GPCRs and non-GPCRs differentiation diagram in this paper is shown by Matplotlab, and the accuracy was not calculated correctly. 2. The universality of this experiment is relatively low. The CTDC method and MRMD2.0 dimension reduction method may only be applicable to GPCRs protein sequence but not to other protein sequence. In the study of Prediction of G protein-coupled sensor, cross validation and Random Forest can be used on other protein sequences (Lai et al., 2018; Tang et al., 2018), especially the proposed framework can be applied to protein fold recognition (Wei et al., 2016; Liu et al., 2017), protein remote homology (Liu et al., 2020), protein subcellular localization (Lv et al., 2019), etc.

## Discussion

Like other macromolecules, proteins are important parts of the living body, the material basis of life, and they participate in almost every activity in the cell. Proteins perform many functions in the body. Through the study of proteins, the mechanism of diseases can be studied, and the design of new drugs can also be promoted. With the advent of machine learning, the function prediction of proteins has also flourished. Obtaining high-performance classification models, accurately and efficiently extracting protein sequences, and converting them into equal-length amino acid sequences have become research directions of many scientists.

Compared with the traditional experimental method, a set of experimental schemes in this paper replaces the redundant experimental steps. Using the CTDC method and dimensionality reduction in CTD, the redundant attributes in the protein sequence features are successfully removed, and they are drawn intuitively using Matplotlib. The division map between GPCRs and non-GPCRs is then drawn. In the division map, there can be a clear distinction between GPCRs and non-GPCRs. This experiment has achieved a certain degree of accuracy.

There are still many aspects that need to be further studied. The Matplotlib coordinate chart used to classify GPCRs and non-GPCRs can only distinguish the relatively large positive and negative samples after being divided by attributes, extracting several solutions: 1. The use of a single Matplotlib coordinate diagram is simple to operate and has many limitations; thus, it cannot reach high accuracy. In the later stage, more comprehensive computational intelligence method such as neural networks (Song et al., 2018a; Zhou et al., 2018; Bao et al., 2019; Hong et al., 2019; Sun et al., 2020), network methods (Sun et al., 2014; Zhou et al., 2015, 2016; Song et al., 2018b; Zeng et al., 2018) and evolutionary strategies (Xu et al., 2019a,b; Zeng et al., 2019b) can be adopted to take the extracted protein features as input. Thus, the positive and negative samples can be divided more accurately, and accuracy can be obtained. 2. In terms of high extraction accuracy, a more comprehensive protein feature extraction method combined with the dimension reduction method (Yang et al., 2019; Zhu et al., 2019) for GPCRs pruning was attempted to screen out features with higher differentiation between GPCRs and non-GPCRs.

## Data Availability Statement

The datasets presented in this study can be found in online repositories. The names of the repository/repositories and accession number(s) can be found in the article/supplementary material.

## Author Contributions

ZC made the design of the subject and the whole idea of the whole experiment in the early stage. XG did comparative experiments and experimental data analysis. DW analyzed the results of the comparative experiment. All authors contributed to the article and approved the submitted version.

## Conflict of Interest

The authors declare that the research was conducted in the absence of any commercial or financial relationships that could be construed as a potential conflict of interest.
